# Accuracy of Resolution of ST-Segment Elevation in Electrocardiogram to Determine the Patency of Infarct-Related Artery

**DOI:** 10.7759/cureus.14448

**Published:** 2021-04-13

**Authors:** Muhammed Kashif Shaikh, Syed Zulfiquar Ali Shah, Chandar Kumar, Munisha Lohano, Abdul Subhan Talpur, Anika Zahoor, Vijay Kumar, Besham Kumar

**Affiliations:** 1 Interventional Cardiology, Liaquat University of Medical and Health Sciences, Jamshoro, PAK; 2 Medicine, Liaquat University of Medical and Health Sciences, Jamshoro, PAK; 3 Cardiology, Liaquat University of Medical and Health Sciences, Jamshoro, PAK; 4 Internal Medicine, United Medical and Dental College, Karachi, PAK; 5 Internal Medicine, Jinnah Postgraduate Medical Centre, Karachi, PAK

**Keywords:** angiography, ecg, patency, infarct-related artery

## Abstract

Introduction: There is very limited data comparing the accuracy of ECG to angiography in predicting reperfusion status. In this study, we will determine the accuracy of ECG change i.e. resolution of ST-segment elevation in predicting infarct-related artery (IRA) patency after thrombolysis in patients with ST-segment elevated myocardial infarction (STEMI), in comparison to angiography.

Methods: Three hundred and forty-one (n = 341) patients with acute STEMI received streptokinase, a thrombolytic agent within 12 hours of symptoms, and were enrolled in the study via consecutive convenient non-probability sampling. ECG was recorded as soon as the patient arrived in the emergency unit of cardiology. Subsequent ECG was recorded three hours after the administration of streptokinase to look for resolution of ST-segment elevation. ST-segment resolution was classified as greater/equal to 50% resolved or less than 50% resolved. Coronary angiography was performed within 24 hours of hospitalization and flow in the IRA was assessed.

Results: The most common site of myocardial infarction (MI) was the anterior wall (50.1%) and the commonest artery involved was the left anterior descending artery (44.2%). On ECG, ST-resolution of more than 50% was found in 242 (70.9%) participants. Thrombolysis in MI (TIMI) grade III flow in angiography was found in 211 (61.8%) participants. The sensitivity and specificity of ST-resolution to detect TIMI grade III flow was 94.79% and 67.69%, respectively, while accuracy was 84.46%.

Conclusion: ST-resolution on ECG after streptokinase can predict IRA patency on coronary angiography with moderate to good accuracy. ECG can assist in predicting the impact of streptokinase early in the course of management and give an option of monitoring patient prognosis with a non-invasive test in patients not comfortable with angiography.

## Introduction

Coronary artery disease is a condition in which there is an inadequate supply of blood and oxygen to the myocardium. It results from occlusion of the coronary arteries and results in a demand-supply mismatch of oxygen. It typically involves the formation of plaques in the lumen of coronary arteries that impede the blood flow [1]. Depending on the level of insult, acute coronary syndrome (ACS) presents in the form of unstable angina, non-ST segment elevation myocardial infarction (NSTEMI) or ST-segment elevation myocardial infarction (STEMI) [2]. In Pakistan, STEMI was responsible for 56% of all myocardial infarction (MI) in a study conducted in 2015 [3].

Management of STEMI recommends reperfusion therapy less than 12 hours after symptom onset [4]. Intravenous thrombolysis is associated with a significant reduction in mortality in acute STEMI [5]. However, in only 60%-70% of patients of acute STEMI, thrombolytic therapy results in grade III flow as per the thrombolysis in MI (TIMI) classification system [6]. Angiography is a gold standard technique to measure the patency of infarct-related artery (IRA) [6]. Non-invasive techniques such as an ECG can also be used to predict the reperfusion status [7]. Ahmed et al. indicated in their pilot study that a 50% reduction in the sum of ST-segment elevations at four hours after intravenous streptokinase was a useful indicator of the outcome of reperfusion [8]. Hogg et al. reported a high sensitivity of 93% in patients with more than 50% resolution of ST-elevation and specificity of 67% [9]. However, very limited data are available comparing the predictive value of ECG in assessing the patency of IRA after thrombolysis.

In this study, we will determine the accuracy of ECG change, i.e. resolution of ST-segment elevation in predicting IRA patency after thrombolysis in patients with STEMI, in comparison to angiography. If proven effective, with the help of ECG we shall be able to monitor the prognosis of patients with a less invasive procedure and at a cheaper cost.

## Materials and methods

This longitudinal study was conducted in the cardiology department of Liaquat University of Medical Health Sciences from August 2020 to January 2021. Ethical approval was taken before the enrollment of patients. Three hundred and forty-one (n = 341) new patients with acute STEMI received streptokinase, a thrombolytic agent within 12 hours of symptoms. Patients were enrolled in the study via consecutive convenient non-probability sampling. STEMI was confirmed based on symptoms, electrocardiogram changes (> 1mm ST-segment elevation) and an increase in cardiac enzymes. Details of the study and risk factors associated with the procedure were explained to the patient or their attendant and consent were taken from them.

ECG was recorded as soon as the patient arrived in the emergency unit of cardiology. A detailed history was taken from the patient and/or attendant. Standard treatment, consisting of oxygen therapy, antiplatelet therapy and analgesics, for STEMI was started immediately. A single dose of 1.5 million international unit (IU) streptokinase was infused intravenously over one hour. Subsequent ECG was recorded three hours after the administration of streptokinase to look for resolution of ST-segment elevation. ST-resolution was classified as either greater than 50% resolved or less than 50% resolved. Coronary angiography was done within 24 hours of hospitalization and flow in the IRA was assessed. Successful thrombolysis was defined as patients having TIMI grade III flow.

Statistical Package for Social Sciences® software version 23.0 (SPSS; IBM Corp., Armonk, NY, USA) was used for data analysis. For numerical variables, data such as age were expressed as mean ± standard deviation. Frequencies and percentages were used for categorical variables, such as gender, comorbidities, family history, site of MI, vessel involved, TIMI grade III flow and ST-resolution. An online calculator (MedCalc, Ostend, Belgium) was used to calculate the sensitivity, specificity, positive and negative predictive value for ST-resolution in predicting the success of thrombolysis.

## Results

The mean age of participants in this study was 49 ± 10 years. History of hypertension and previous MI was found in 53.3% and 26.6% participants, respectively (Table 1).

**Table 1:** Demographics and baseline clinical characteristics of the patients

Characteristics	Frequency (Percentage %)
Age in years (Mean ± Standard Deviation)	49 ± 10
Gender	
Male	200 (58.6%)
Female	141 (41.4%)
Known History of Comorbidities	
Hypertension	182 (53.3%)
Type 2 Diabetes Mellitus	162 (47.5%)
Hyperlipidemia	151 (44.2%)
Smoker	112 (32.8%)
Hyperuricemia	52 (15.2%)
Past and Family History	
Family History of Myocardial Infarction	91 (26.6%)
Previous History of Myocardial Infarction	21 (6.1%)

The most common site of MI was the anterior wall (50.1%) and the commonest artery involved was the left anterior descending artery (44.2%) (Table 2).

**Table 2:** Site of myocardial infarction and the artery involved

Site of Myocardial Infarction	Frequency (Percentage %)
Wall Involved	
Anterior Wall Myocardial Infarction	171 (50.1%)
Inferior Wall Myocardial Infarction	99 (29.0%)
Left Wall Myocardial Infarction	71 (20.8%)
Artery Involved	
Left Anterior Descending	151 (44.2%)
Right Coronary Artery	89 (26.0%)
Left Circumflex Artery	81 (23.7%)
Left Coronary Artery	20 (5.8%)

On ECG, ST resolution of more than 50% was found in 242 (70.9%) participants. TIMI grade III flow in angiography was found in 211 (61.8%) participants. The sensitivity of ST-resolution to detect TIMI grade III flow was 94.79%, specificity was 67.69% and accuracy was 84.46% (Table 3).

**Table 3:** Outcome on the basis of ST-resolution ECG: electrocardiogram, TIMI: Thrombolysis in Myocardial Infarction, PPV: positive predictive value, NPV: negative predictive value

ECG ST-Resolution		TIMI Grade III Flow		Sensitivity % (CI)	Specificity	PPV	NPV	Accuracy
		Yes	No					
More than 50%	242	200	42	94.79% (90.86% to 97.37%	67.69% (58.93% to 75.63%)	82.64% (78.75% to 85.95%)	88.89% (81.64% to 93.50%)	84.46% (80.17% to 88.14%)
Less than 50%	99	11	88					

## Discussion

The most common comorbidity reported by the participants with acute STEMI was hypertension (53.3%), followed by type 2 diabetes mellitus (47.5%). Diabetes is a global problem, and adult diabetes is expected to increase from 2.8% in 2000 to 4.4% in 2030 [10]. A positive family history of MI was recorded in 26.6% of the patients; which is consistent with a study conducted by Saleem et al. [11]. In assessing the most common wall involved in MI, anterior wall MI (50.1%) topped the list, followed by inferior wall MI (29.0%). These trends have been supported by a study carried out by Saleem et al. [11]. In terms of artery involvement, the left anterior descending was reported to be affected among 44.2% of the patients, followed by right coronary artery involvement (26.0%).

Our study monitored ECG three hours after the administration of streptokinase to measure the extent of the resolution of ST-segment elevation. After the administration of streptokinase, ST-resolution of more than 50% was observed in 70.9% of the patients. Effective results have been observed in a similar study conducted at Hayatabad Medical Complex, Peshawar; both partial and complete ST-segment resolution was observed on ECG in 77.11% patients, three hours after the administration of streptokinase [11]. However, the study also focused on the fact that administration of thrombolytic within 12 hours has shown better results. The difference in results depending on the hourly administration of streptokinase has also been proven by Ch et al. [12]. TIMI grade III flow in angiography was reported in 61.8% participants.

Another study monitored ST resolution on ECG one hour after streptokinase administration, 64% patients showed ST resolution, the study also proved that patients who did not show ST resolution were comparatively at a higher risk of developing complications [13]. It is of keen importance to repeat ECG at regular intervals for better insight into the prognosis as ST resolution improves with time. Anderson et al. assessed the efficacy of thrombolytic therapy on ST resolution in subsequent ECG. ST resolution was reported in 44.2% and 56.5% of the patients after one and a half hours and three hours, respectively [14]. The finding of this study is significant, as it provides a non-invasive option in contrast to the coronary angiogram that might not only make the patient anxious but may also be associated with complications [15].

To the best of our knowledge, this is the first study in the local population that compares ECG with angiography to predict the outcome of thrombolysis. Because of the high sensitivity of ECG found in our study, it would give an option to a physician to regularly monitor prognosis without any invasive procedure. However, since our study was conducted in one center, the sample size may be less diverse. Large-scale multicenter studies are needed to confirm finding of our study.

## Conclusions

In patients presenting with acute STEMI, ECG being a simple, readily available, non-invasive and economical modality can be used repetitively as needed for the timely assessment of the quality of reperfusion therapy. ST-resolution on ECG after the administration of streptokinase can predict IRA patency with moderate to good accuracy. Owing to its high sensitivity, ECG can not only assist in predicting the impact of streptokinase early in the course of management but also prove to be a useful tool for risk stratification. Hence, high-risk patients may be promptly referred for an invasive evaluation. Conversely, a low-risk patient can be successfully identified and an immediate adjunctive coronary intervention can safely be avoided; therefore preventing unnecessary intervention and distress to the patient.
